# Functional cooperation between FACT and MCM is coordinated with cell cycle and differential complex formation

**DOI:** 10.1186/1423-0127-17-11

**Published:** 2010-02-16

**Authors:** Bertrand Chin-Ming Tan, Hsuan Liu, Chih-Li Lin, Sheng-Chung Lee

**Affiliations:** 1 Department of Life Science, College of Medicine, Chang Gung Univeristy, Taoyuan, Taiwan; 2 Institute of Molecular Medicine, College of Medicine, National Taiwan University, Taipei, Taiwan; 3 Institute of Biological Chemistry, Academia Sinica, Taipei, Taiwan

## Abstract

**Background:**

Functional cooperation between FACT and the MCM helicase complex constitutes an integral step during DNA replication initiation. However, mode of regulation that underlies the proper functional interaction of FACT and MCM is poorly understood.

**Methods & Results:**

Here we present evidence indicating that such interaction is coordinated with cell cycle progression and differential complex formation. We first demonstrate the existence of two distinct FACT-MCM subassemblies, FACT-MCM2/4/6/7 and FACT-MCM2/3/4/5. Both complexes possess DNA unwinding activity and are subject to cell cycle-dependent enzymatic regulation. Interestingly, analysis of functional attributes further suggests that they act at distinct, and possibly sequential, steps during origin establishment and replication initiation. Moreover, we show that the phosphorylation profile of the FACT-associated MCM4 undergoes a cell cycle-dependent change, which is directly correlated with the catalytic activity of the FACT-MCM helicase complexes. Finally, at the quaternary structure level, physical interaction between FACT and MCM complexes is generally dependent on persistent cell cycle and further stabilized upon S phase entry. Cessation of mitotic cycle destabilizes the complex formation and likely leads to compromised coordination and activities.

**Conclusions:**

Together, our results correlate FACT-MCM functionally and temporally with S phase and DNA replication. They further demonstrate that enzymatic activities intrinsically important for DNA replication are tightly controlled at various levels, thereby ensuring proper progression of, as well as exit from, the cell cycle and ultimately euploid gene balance.

## Background

Complete and precise DNA replication is essential to the maintenance of genomic integrity and balance. Initiation is the most critical regulatory step, which coincides with the onset of S phase and requires prior assembly of pre-replicative complexes (preRCs). Reinitiation of DNA replication is usually prevented, and only a single round of DNA duplication is performed in a cell cycle. Such restriction mechanism, called replication licensing, partly lies in the regulation of preRC assembly. The protein components of the preRC complex include origin recognition complex (ORC), Cdc6, Cdt1 and minichromosome maintenance proteins (MCM2-7). Phosphorylation of components of the assembled pre-RC constitutes a second level of initiation regulation, upon which the initiation of DNA replication is triggered at the G_1_-S boundary [1-3]. Finally, as with the formation of pre-RC, the transition to DNA replication involves the association of additional replication factors that facilitate unwinding of the origin DNA, as well as multiple DNA polymerases [4]. Following origin activation, new DNA synthesis begins as replication forks move away from the initiation region [1,5,6].

Among different replication factors, the hexameric helicase complex MCM provides an essential activity, catalyzing the unwinding of DNA duplex [7]. Previous work has established a direct role of MCM in not only the initiation step, but also the elongation stage of DNA replication [4,8]. MCM possesses various functional features that are coordinated with other events of the cell cycle [1,7]. Consistent with its functional significance, several regulatory mechanisms have been uncovered that serve to preserve and restrict its proper activities [9]. Phosphorylation accounts for a major regulation. Activation of the MCM complex requires the actions of both the CDC7/DBF4 and cyclin-dependent kinases [1,2]. Mitotic and DNA damage-induced phosphorylation of the MCM4 subunit, concomitant with loss of activity and/or subcellular localization change, involves CDK2-cyclin A or cyclin B [10-14]. Another mode of regulation lies in the combinatorial formation of MCM subassemblies. Aside from the expected heterohexameric complex (MCM2/3/4/5/6/7), *in vitro *experiments have demonstrated the formation of several stable subassemblies including MCM2/4/6/7, MCM4/6/7, and MCM3/5 complexes [15-18]. Among them, a weakly processive DNA helicase activity was identified in the MCM4/6/7 complexes of human, mouse, and fission yeast, whereas the heterohexamer lacks such activity [15,16,19,20]. Work done by Schwacha and Bell further discriminated two functionally distinct MCM protein subgroups: the "catalytic core" MCM4/6/7 and the "regulatory" MCM2p, 3p, 5p [21]. These results suggest that distinct assemblies of MCM subunits may contribute optimally to the coordinated and differential actions during the progression of replication.

Chromatin poses yet another type of regulation of the MCM activity, and the progression of replication in general, in an inhibitory fashion [1,22]. Various reports have shown that local chromatin environment, as well as chromatin remodeling factors, directly dictates activity of the replication origin and DNA replication [23-28]. As demonstrated by our recent work, nucleosomes impose a structural hindrance that efficiently reduces the DNA helicase activity of MCM [29]. Functional interaction between MCM and the FACT heterodimeric complex, however, alleviates such inhibition and concomitantly facilitates chromatin DNA unwinding. Our findings, together with those from other groups, show that the FACT-MCM complex plays an important role in the normal progression of DNA replication initiation and S phase *in vivo *[29-31]. Mode of regulation for this essential chromatin unwinding activity has not been characterized presently.

In this study we present several lines of evidence linking the functions of the FACT-MCM complex to cell cycle progression and regulation. Functional attributes such as origin association, DNA unwinding activities, and complex formation are intimately coordinated with cell cycle progression. Furthermore, the FACT-MCM interaction is generally dependent on persistent cell cycle and cell proliferation. These results suggest that the FACT-MCM complexes are tightly controlled at various levels to safeguard its essential activity as well as precise DNA synthesis.

## Methods

### Antibodies and Western blot analysis

Generation of monoclonal antibodies against SSRP1 (2B12/control, 10D1, and 10D7) and hSpt16p (8D2) was described previously [32]. Anti-human ORC1 monoclonal antibody was purchased from NeoMarker/Lab Vision. Polyclonal serum against human pan-MCM was purchased from BD biosciences. Monoclonal antibody against Cdc6 and rabbit antisera against MCM3, MCM4, MCM5, and MCM6 were produced with the following peptide antigens and affinity purified by Dagene (Taiwan). Cdc6: MCM3: SDTEEEMPQVHTPKTAD; MCM4: SRRGRATPAQTPRSED; MCM5: KEVADEVTRPRPSGE; MCM6: KYLQLAEELIRPERNT. Polyclonal antibody against MCM2 was generated using a recombinant protein fragment of MCM2 (a.a. 792-892). Monoclonal antibody against Cdc45 (E-3) was purchased from Santa Cruz Biotechnology. Anti-FLAG mAb (M2) was purchased from Sigma. Western blot analysis was performed after electrophoretic separation of polypeptides by 7.5% or 10% SDS-PAGE and transfer to Hybond-C membranes. Blots were probed with the indicated primary and appropriate secondary antibodies, and detected by ECL chemiluminescence (Amersham).

### Immunoprecipitation

HeLa or K562 cells were extracted using a buffer containing: 20 mM HEPES (pH 7.4), 0.2 M NaCl, 0.5% TX100, 5% glycerol, 1 mM EDTA, 1 mM EGTA, 10 mM β-glycerophosphate, 2 mM Na_3_VO_4_, 1 mM NaF, 1 mM DTT, plus protease inhibitors. For preparation of nuclear extracts, HeLa nuclei were isolated and lysed in nuclear extraction buffer (10 mM HEPES with pH 7.9, 10 mM KCl, 0.1 mM EDTA, 0.1 mM EGTA, 0.1% TX-100, 0.4 M NaCl, 10% glycerol and protease inhibitors). All immunoprecipitations were done with the indicated antibodies prebound to protein G-Sepharose (Amersham), and washed in the cell lysis buffer.

### Gel-filtration fractionation

Gel filtration chromatography was done using a precalibrated Sephacryl S-400 HR column with a bed volume of 135 ml (Pharmacia). Nuclear lysate preparation, chromatographic settings, and fraction collection and processing were done essentially as reported previously [32].

### Cell culture and cell cycle analysis

All HeLa cells were maintained in Dulbecco's modified Eagle's medium supplemented with 10% fetal bovine serum and 100 units/ml penicillin and streptomycin. Cells were transfected using Lipofectamine (GIBCO) according to the manufacturer's instructions. For monitoring cell cycle progression and immunostaining analysis, collection of HeLa cells at different stages of the cell cycle was achieved by the mitotic shake-off or the double thymidine block method, as outlined previously [32]. Procedure for the FACS analysis was also described in the same report. Early G_1 _cells were collected by replating mitotic cells and allowing attachment for 2-4 hrs. To achieve a differentiation (or resting) phase in K562 cells, cells were subjected to 3-day treatment of 2 mM sodium butyrate [33].

### Plasmid-based dsRNAi

To establish a plasmid-based dsRNAi system targeting endogenous MCM3 or MCM4, annealed oligonucleotides corresponding to partial sequence were designed and ligated to the pSuper.neo+GFP (OligoEngine) according to the manufacturer's instructions. The cDNA sequence of the targeted mRNA region for different genes is as follows. MCM3: 5'-AAACGAGAAGAGGGCTAAC-3' (nucleotides 171-189); MCM4: 5'-GACACCACACACAGTTATC-3' (nucleotides 1095-1113). The same sequence in the inverted orientation was used as the non-specific dsRNAi control.

### Indirect immunofluorescence and confocal microscopy

All steps of the immunostaining procedure were essentially identical to a previous report [32]. Subsequent to staining with the indicated primary antibodies, secondary antibody incubation was done for 1 h using Alexa 488 conjugated goat anti-mouse IgG and Alexa Fluor 594 conjugated goat anti-rabbit IgG (Moleular Probes, Inc). To visualize DNA, cells were counter-stained with DAPI. Stained cells were analyzed with the Zeiss LSM-510 inverted confocal laser-scanning microscope, using a 100X/NA 1.4 oil immersion or 40× objective lens.

### DNA helicase assay

The substrate for the helicase assay was a partially heteroduplex DNA containing a 17-mer oligonuleotide (5'-GTTTTCCCAGTCACGAC-3') annealed to the M13mp18 (+) circular ssDNA (Amersham). Before annealing, the oligonuleotide was labeled at the 5' end with [γ-^32^P]ATP by polynucleotide kinase. The annealed substrate was subsequently purified on the MicroSpin G50 column (Amersham). MCM-containing FACT immunocomplexes were isolated by the indicated antibodies and from HeLa cells at the specified cell cycle stages. With the exception of the immobilized source of enzymatic activities, DNA helicase assay was performed essentially as described previously [34], with the addition of approximately 10-20 fmol of substrate. After deproteination, samples were resolved by electrophoresis (15% native PAGE/TBE) and autoradiographed.

### Chromatin immunoprecipitation

Chromatin immunoprecipitation assays were modified from previously described methods [35]. Briefly, HeLa cells (exponentially growing or synchronized) were cross-linked with 1% formaldehyde for 10 min at 37°C. The nuclei were isolated and sonicated into oligonucleosomes of ~500-600 bp in length. The sheared chromatin was immunoprecipitated overnight with protein G-agarose previously bound with the 10D1, 8D2, or control antibody. After extensive washes, the immunoprecipitates were subjected to deproteination and cross-linking reversal. The presence of genomic DNA in the precipitates was detected by PCR with the B48 primer set and a background primer set. The background primers anneal to a region with no annotated genes, 30 kb upstream of the lamine B2 origin sequence on chromosome 19, and have the following sequences: 5'-CTATGCCAAGCCCATTCTAGGTCCT-3 (sense); 5'-GCAGGGAAACTGTGCACAGCAAGAG-3' (antisense). Upon amplification for 27-30 cycles, the products were resolved by 2% agarose gels and visualized with ethidium bromide staining. UV-illuminated images were photographed and analyzed by AlphaImager 1220 (Alpha Innotech Corp.)

## Results

### Subassemblies of MCM form distinct complexes with FACT heterodimer

**Figure 1:**
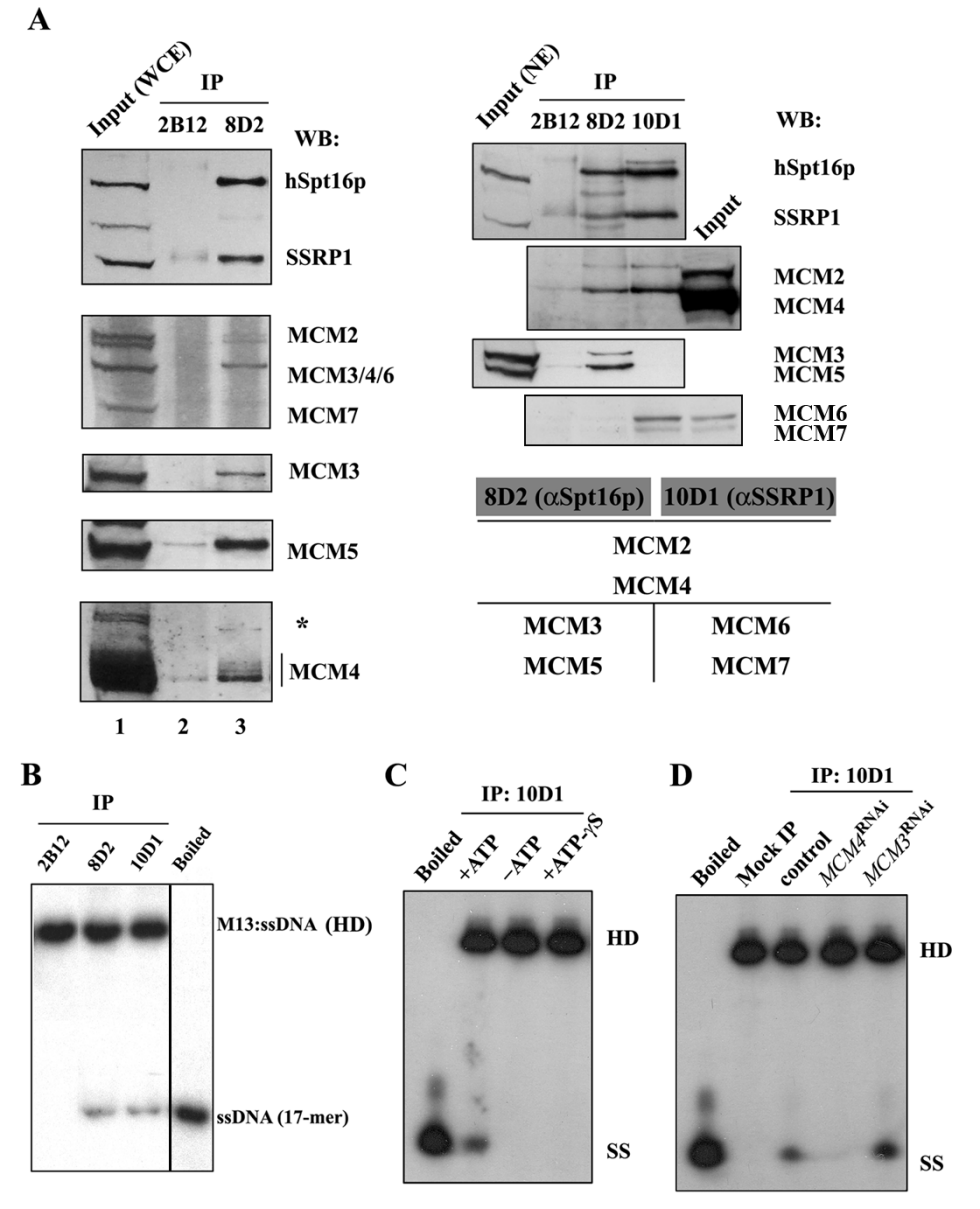
**Subassemblies of MCMs form distinct, DNA unwinding-competent complexes with FACT**. (A) Western blot analysis of HeLa whole cell extracts (left panel) and nuclear extracts (right panel), as well as the different immunocomplexes targeted by control (2B12), α hSpt16p (8D2), and α SSRP1 (10D1) mAbs. Immunoblotting was done using the indicated antibodies against pan-MCM or individual subunits. The amount of the Input is equivalent to 1/40 the IP. The identity of the protein band, marked by the asterisk, is unknown. The chart summarizes the constituents of the different FACT immunocomplexes. (B) Immunocomplexes were isolated as in (A) and subjected to DNA helicase assay. The reaction was conducted on a radiolabeled, 17-mer oligonucelotide annealed on the M13 single-stranded DNA ("HD", heteroduplex DNA). Upon protein removal, reaction mixtures were resolved by native gel. The locations of the annealed and displaced (ssDNA) substrates on the gel are shown. Displacement of the annealed substrate by heat denaturation is also shown (Boiled). (C) The DNA helicase activity of the FACT-MCM complex is ATP-dependent. The displacement of 17-mer oligonucleotide (SS) from the heteroduplex substrate (HD) by the 10D1-immunocomplex was assayed in the presence (lane 2) or absence (lane 3) of ATP, or in the presence of ATP-γ S (lane 4). (D) HeLa cell extracts were prepared from control (lanes 2 & 3), *MCM4*^RNAi ^(lane 4), or *MCM3*^RNAi ^(lane 5) cells. The displacement activity of the mock- (lane 2) or 10D1- (lanes 3-5) immunocomplex isolated from these extracts is shown.

Our previous work on the transcription elongator FACT has identified and characterized the physical and functional interaction between FACT and a subassembly of the MCM helicase complex, namely MCM2/4/6/7 [29]. To carry out a more thorough examination of FACT-associated complexes, we performed additional Western analysis on different anti-FACT immunoprecipitates isolated from whole cell (Figure 1A, left panel) and nuclear extracts (right panel). The specificity of these antibodies was demonstrated by the lack of cross-reactivity to MCM proteins (Additional file 1 Figure S1A). Intriguingly, by using different antibodies that recognize pan or individual MCM subunits, we discovered that distinct subsets of MCM proteins are associated with the anti-hSpt16p (8D2) or anti-SSRP1 (10D1) precipitates (as summarized in a chart in Figure 1A, bottom right panel). Whereas both MCM2 and MCM4 are commonly shared between these two types of complexes, differential coprecipitation of MCM3/5 (by 8D2) and MCM6/7 (by 10D1) was observed (Figure 1A). These results demonstrate that FACT may interact with discrete subcomplexes of the MCM proteins.

The primary biochemical activity of the MCM complex is the unwinding of the DNA strands [15,19]. Thus, to examine whether the identified MCM-associated FACT complexes are catalytically active, we performed DNA helicase assay. Immunoprecipitates were incubated with labeled substrate and their abilities to displace annealed oligonucleotide were assayed. We found that, as compared to the control antibody, FACT complexes precipitated by either 8D2 or 10D1 antibody possessed DNA helicase activity (Figure 1B). This suggests that the two distinct FACT immunocomplexes, although differing in MCM constituents, are both competent in unwinding DNA *in vitro*. Furthermore, such catalysis is an ATP-dependent process, as no such activity was detected in the absence of ATP, nor in the presence of nonhydrolyzable form of ATP (Figure 1C).

To further characterize whether the DNA helicase activity displayed by the FACT immunocomplexes is mediated through the associated MCM complex, we isolated from *MCM4*^RNAi ^cells (Additional file 1 Figure S1B) the 10D1 immunoprecipitates that are deficient in the MCM complex and found that the DNA helicase activity was greatly reduced (Figure 1D). This reduction was not observed in the 10D1 immunoprecipitates isolated from control or *MCM3*^RNAi ^cells (Additional file 1 S1B) in which the FACT-MCM association is still present. This result serves as strong evidence that the helicase activity of the 10D1 immunocomplexes can be attributed primarily to the associated MCM complex, but not any non-specifically associated activities.

**Figure 2:**
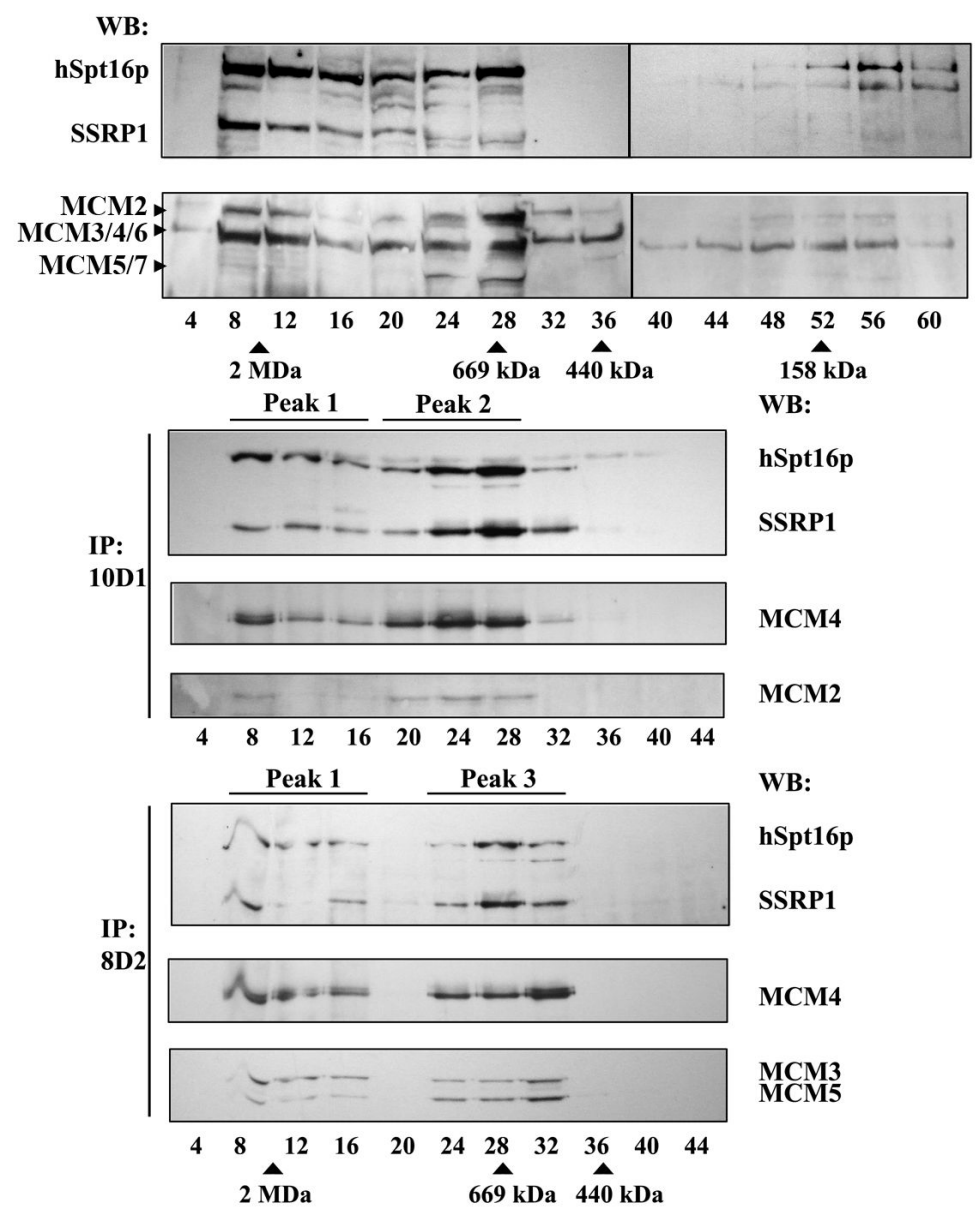
**Fractionation profile of distinct FACT-MCM complexes**. HeLa nuclear extracts (Figure 1A) were subjected to gel filtration chromatography using Sephacryl S-400. Generated fractions (numbers as indicated) were immuno-probed with the specified antibodies. Selected fractions were further subjected to immunoprecipitation using 10D1 and 8D2, and subsequently probed with the indicated antibodies. Peaks 1, 2, and 3, as shown in the 10D1 IP and 8D2 IP panels, denote the approximate fractions in which copurification of MCM with FACT was observed.

Next, we subjected HeLa nuclear extracts (Figure 1A) to gel filtration to further verify the presence of FACT-MCM complexes. As shown by Western blot, both FACT and MCM subunits have broad and overlapping distributions in fractions ranging in molecular size from 669 kDa to 2 MDa (Figure 2). To further distinguish the physical association between the FACT and MCM subcomplexes, we subjected gel filtration fractions to immunoprecipitation. Presence of the MCM subunits in the precipitates isolated by the two antibodies (8D2 and 10D1) from fractions 8-32 confirms the coelution of these two complexes and suggests that they combine to form complexes of various sizes (Figure 2). Furthermore, this immunoprecipitation assay approximately resolved three different peaks of MCM copurification (Figure 2, lower section). Interestingly, we could observe a shift in peak elution between peak 2 (10D1) and peak 3 (8D2), indicating a difference in the apparent sizes of FACT-MCM2/4/6/7 and FACT-MCM2/3/4/5. The MCM constituents of these immunocomplexes, as shown by Western blot, are consistent with the above immunoprecipitation experiments that detected two separate types of FACT-MCM association (Figure 1A). Together, these observations strongly corroborate the existence of two distinct FACT-MCM subcomplexes. The true identity of the immunocomplexes in peak 1 fractions cannot be deduced by our experiment. In summary, our biochemical analyses definitely characterize two native, enzymatically active FACT-MCM complexes that contain either the MCM2/4/6/7p or the MCM2/3/4/5p subassembly with the common partner FACT. Our results do not preclude, however, the existence of additional, higher molecular-weight FACT-MCM complexes (in the MDa range) that might include other interacting polypeptides or DNA.

### Functional attributes of the two distinct FACT-MCM sub-complexes

**Figure 3:**
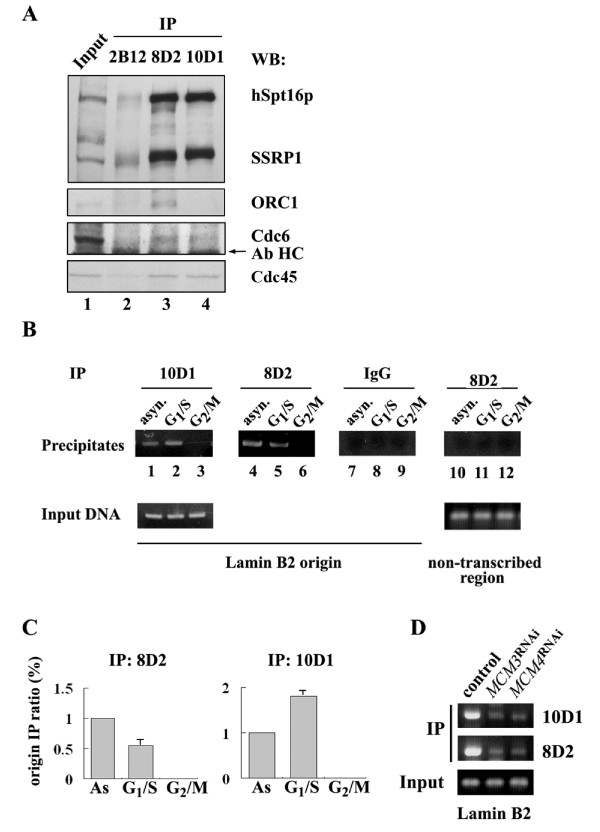
**Functional attributes of the two distinct FACT-MCM assemblies**. (A) HeLa WCE was subjected to immunoprecipitation with a control (lane 2), 8D2 (lane 3), or 10D1 (lane 4) antibody. Lane 1 is the extract input for the IP (1/40). Existence of pre-RC components (ORC1 and Cdc6) and Cdc45 in these immunoprecipitates was detected by specific antibodies. (B) ChIP was performed as described in Methods. Sonicated chromatin fragments were prepared from cells at different stages: asynchronous (lanes 1, 4, 7 & 10), G_1_/S (lanes 2, 5, 8 & 11), and G_2_/M (lanes 3, 6, 9 & 12). Immunoprecipitation was done with either control (IgG, lanes 7-9), 8D2 (lanes 4-6, 10-12), or 10D1 (lanes 1-3) antibody. Products from final PCR analysis using primers specific to lamin B2 origin (lanes 1-9) or to a non-transcribed region (lanes 10-12) were resolved by 1.5% agarose gel. Precipitated products are shown in the upper panels, DNA input in the lower. (C) Degree of origin association of the two immunocomplexes was quantitatively determined by the normalized ratio of amplified origin sequence, with the ratio of the asynchronous immunoprecipitate represented as 1. The histograms summarize such calculation and compares the origin binding of the FACT immunocomplex (8D2, left; 10D1, right) at each cell cycle stage. Data are averaged ± standard deviations of three independent experiments. (D) ChIP was performed as in (B). Sonicated chromatin fragments were prepared from control, *MCM3*^RNAi^, and *MCM4*^RNAi ^cells (HeLa). Immunoprecipitation was done with either 8D2 (top panel) or 10D1 (middle panel) antibody. Presence of origin fragment was detected by PCR. PCR products from input DNA are shown in the bottom.

Both FACT-MCM immunocomplexes are equally active in unwinding DNA template, indicating that their distinction may lie in other functional aspect. Thus, to further delineate and discriminate the functional attributes of the FACT-MCM complexes, we first sought to probe for the potential association of other pre-replication complex (pre-RC) factor. To this end, we performed co-immunoprecipitation experiments using the 8D2 and 10D1 antibodies. Endogenous ORC1 and Cdc6 were detected in the 8D2 immunocomplexes (Figure 3A). On the other hand, ORC was absent and the signal of Cdc6 was weak in the 10D1 immunoprecipitates. Such preferential association of the FACT-MCM2/3/4/5 complex with the origin-bound pre-RC may imply its role during pre-RC assembly. We also probed for the presence of Cdc45, which is a component of the large replisome progression complex (RPCs) that also includes MCMs and FACT [31]. Interestingly, we were able to detect this factor in both immunocomplexes (Figure 3A), suggesting that identified FACT complexes may be associated with active replication.

To further evaluate the replicative role of these subcomplexes, we examined their origin association. In our previous work, we discovered that the 10D1-targeted FACT complex is present *in vivo *at a region of known replication origin, namely the replicator associated with the human lamin B2 gene. Using the ChIP assay, chromatin prepared from cells synchronized at different cell cycle stages was precipitated with the 8D2 or 10D1 antibody (Figure 3B). PCR reactions using specific sets of primers were subsequently performed to monitor the existence of the lamin B2 origin sequence (Figure 3B, lanes 1-9). As shown in Figure 3B, at equal loads of chromatin preparations, we observed specific occupancy of 8D2-targeted FACT complex in the ori region in asynchronously growing cells (compare lanes 4 and 7). As a control, sequence of a distant non-transcribed region (Figure 3B, lanes 10-12, see Methods) was not enriched in the immunoprecipitates. Furthermore, no origin binding of either immunocomplexes could be detected in mitotic cells (lanes 3 and 6, and ref. 28). This result is reminiscent of the observed dissociation of FACT from condensed chromatin during mitosis [32]. Interestingly, while the 10D1-complex exhibited an enrichment of such origin binding during the transition from G_1 _to S phase (compare lanes 1 and 2; Figure 3C, histogram on the right), the 8D2-associated complex seemed to display a relatively greater degree of association in G1 as compared to the point entering S phase (lanes 4 and 5; Figure 3C, histogram on the left). These results reflect the activity of both FACT-MCM complexes locally at origins or replication forks, and imply their differential origin association.

Next, to further delineate the differential origin binding modes of the two FACT-MCM immunocomplexes, we performed additional ChIP assays on *MCM3*^RNAi ^or *MCM4*^RNAi ^cells. Chromatin preparations from these two cell lines were subjected to ChIP using either the 8D2 or 10D1 antibody and probed for the presence of the lamin B2 origin sequence (Figure 3D). In the context of downregulated MCM3 or MCM4 protein level, association of the 8D2 immunocomplex with the origin was disrupted (Figure 3D, middle panel). This signals that the origin binding of FACT (and the FACT-MCM2/3/4/5) lies, at least in part, in the presence of an intact MCM2/3/4/5 complex. Furthermore, in the *MCM4*^RNAi ^cells, we saw a similar reduction of the origin fragment in the 10D1 immunocomplex, demonstrating the importance of MCM4 in this functional regard (top panel). Intriguingly, knockdown of the MCM3 subunit unexpectedly led to a weakened association of FACT-MCM2/4/6/7 with lamin B2 origin. A likely explanation for this phenomenon is that the origin association of the MCM3-associated complexes (including FACT-MCM2/3/4/5) may be preceding and required for the subsequent recruitment of FACT-MCM2/4/6/7. Therefore, there may exist two different temporal modes through which the FACT-MCM complexes are recruited to the replicator (see Discussion). Collectively, results presented in Figure 3 suggest that these two FACT-MCM complexes may operate at distinct stages during the initiation phase of DNA replication.

### Functional regulation of the FACT-MCM complex mirrors cell cycle progression

**Figure 4:**
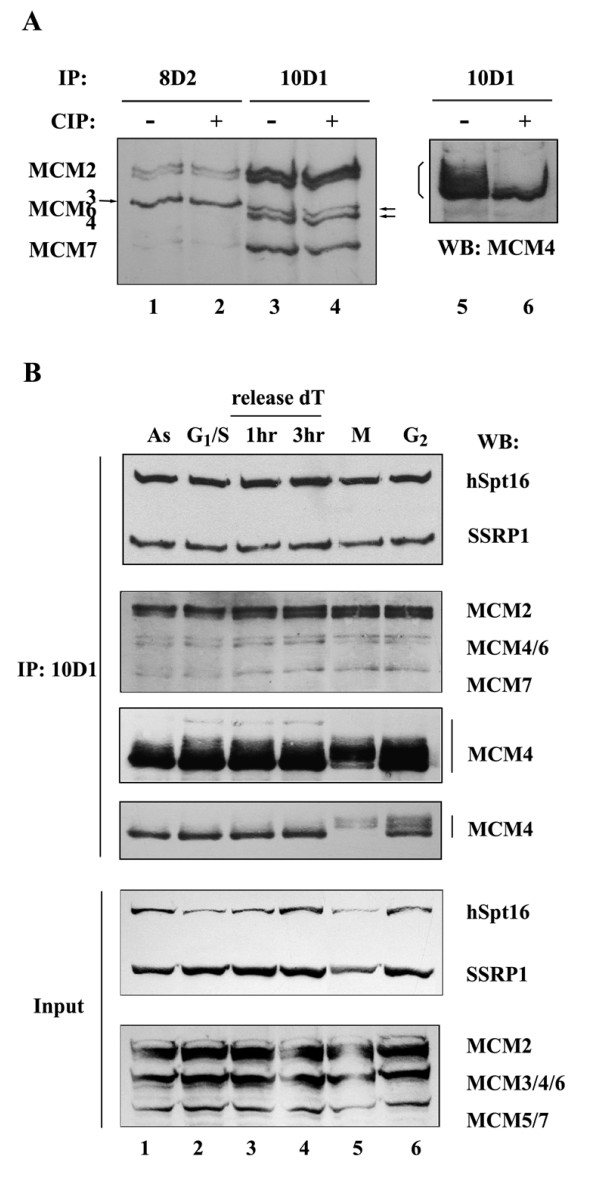
**FACT-associated MCM4 undergoes a cell cycle-dependent phosphorylation change**. (A) HeLa cells were immunoprecipitated with mAb 8D2 (lanes 1 and 2) and 10D1 (lanes 3-6). Immunoprecipitates were further subjected to buffer (lanes 1, 3, and 5) or alkaline phosphatase treatment (lanes 2, 4, and 6). The presence of MCM proteins was detected by Western blot analysis using anti-pan MCM (lanes 1-4) and α MCM4 (lanes 5 and 6) antibodies. The positions of MCM 3/4/6 are distinguished by arrowheads. The smear of immunoreactive signlas for MCM4 is also highlighted. (B) FACT-associated immunocomplex was isolated by α SSRP1 mAb 10D1 from HeLa cells at different cell cycle stages: exponentially growing (lane 1), G_1_/S phase (lane 2), S phase at 1 hr after double-thymidine block (lane 3), S phase at 3 hr (lane 4), and M phase (lane 5) and G_2 _(lane 6). The presence of MCMs and FACT heterodimers was detected by the specified antibodies. The top four panels are immunoblots for the immunoprecipitates, and the bottom two panels are for the lysate inputs. The two anti-Mcm4 blots are from different exposure lengths of signal development (longer exposure on top).

While probing for the presence of MCM4 in the anti-FACT immunocomplexes, we observed smear of immunoreactive signals representative of multiple lower-mobility protein forms (Figures 1A &4A). Changes in electrophoretic mobility of MCM4 may potentially be induced by phosphorylation, as demonstrated previously [11]. Indeed, treatment of the immunoprecipitates with alkaline phosphatase eliminated the higher protein forms (Figure 4A, compare lanes 5 and 6). However, no phophatase-induced electrophoretic mobility change was seen in the other MCM subunits, implying their lack of modification by phosphate-moiety (Figure 4A, lanes 1-4). Importantly, other groups have reported that, in logarithmically growing or S phase HeLa cells, lower mobility form of MCM4 could be detected largely in the chromatin fraction but not in the Triton-soluble fraction [13,14]. Our results therefore suggest that the isolated FACT-MCM complex is likely to exist endogenously in a chromatin-bound form.

**Figure 5:**
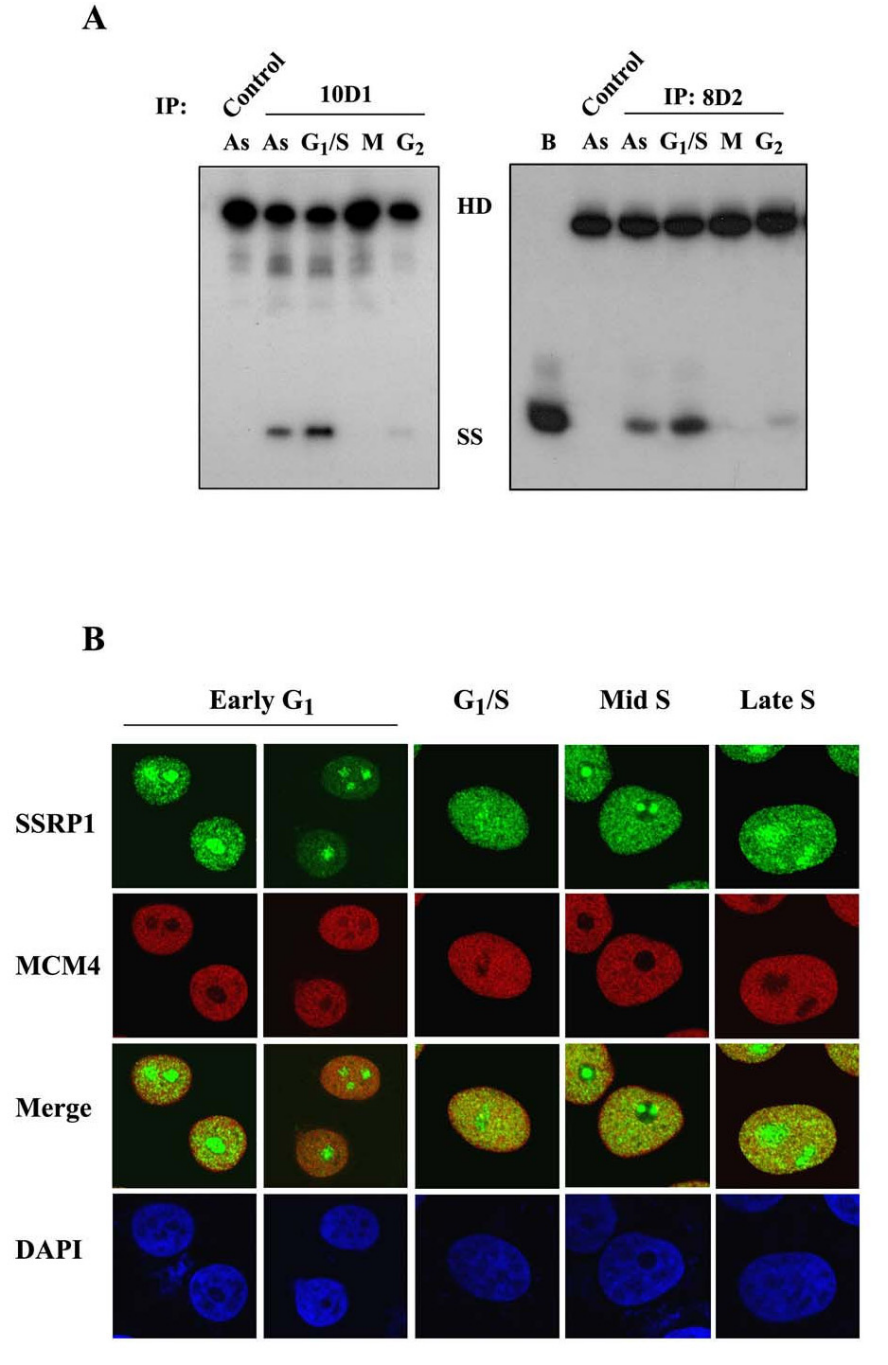
**Catalysis and formation of the FACT-MCM complex is regulated in a cell cycle-dependent manner**. (A) FACT-associated immunocomplexes were isolated by mAbs 10D1 (left panel) and 8D2 (right panel) from HeLa cells at different cell cycle stages: exponentially growing (As), G_1_/S phase, and M phase and G_2_. The immunoprecipitate was subsequently subjected to DNA helicase assay as in Figure 1B. Displacement of the annealed substrate by heat denaturation is also shown ("B"). (B) Indirect immunofluorescence analysis was performed on HeLa cells to observe localization of endogenous MCM4 and SSRP1 using the respective antibodies. Nuclear DNA was counter-stained by DAPI. Cells in indicated phases of the cell cycle were examined: early G_1_, G_1_/S, mid- and late- S phase (cells at 2 and 4 hours after G_1_/S release, respectively). Images were generated by laser scanning confocal microscopy.

Based on the previous observation that mouse MCM4 undergoes mitotic hyper-phosphorylation and functional inactivation [11], we wanted to examine whether the FACT-associated MCM4 (and MCM complex) is similarly modulated in a cell cycle-dependent manner (Additional file 1 Figure S1C). First, at the post-translational modification level, lower-mobility forms of FACT-bound MCM4 became increasingly visible starting in G_2 _phase and peaking in M phase, during which the unmodified form was only marginally present (Figure 4B, middle two panels). While the degree of phosphorylation of MCM4 altered, the profiles of the FACT dimers and the associated MCM subunits remain relatively invariable across cell cycle (Figure 4B, upper two panels). Second, to address whether this alteration in modification status of MCM4 reflects cell cycle-dependent functional changes for the FACT-MCM complex, we performed DNA helicase assay on immunoprecipitates isolated from different stages (Figure 5A). We found that, at the equivalent levels of co-precipitated MCMs (Additional file Figure S1D), the DNA unwinding activity of either the 8D2 or 10D1 immunocomplex peaked at the G_1_/S junction (Figure 5A). However, both FACT-MCM complexes exhibited significantly lower helicase activity during G_2 _and mitosis, similarly to the cell cycle regulation of the core MCM helicase activity [11]. Taken together, our observations are in agreement with the previous results on mouse MCM4. Therefore, we conclude based on these data that the association of FACT with the MCM complex preserves MCM helicase activity and its temporal regulation during cell cycle progression.

Further evidence that links the possible role of FACT-MCM to S phase was obtained from immunofluorescence analysis. While examining the subcellular localizations of these two factors at different cell cycle stages, we found a discernible degree of colocalization between FACT and MCM4 at G_1_/S junction as well as throughout S phase (Figure 5B). However, this colocalization was markedly reduced in early G_1 _(Figure 5B) or G_2_/M cells (data not shown). Such manner of interaction between complexes may contribute to their coordinated functions during S phase.

### Cell proliferation underlies the interaction between FACT and MCM

**Figure 6:**
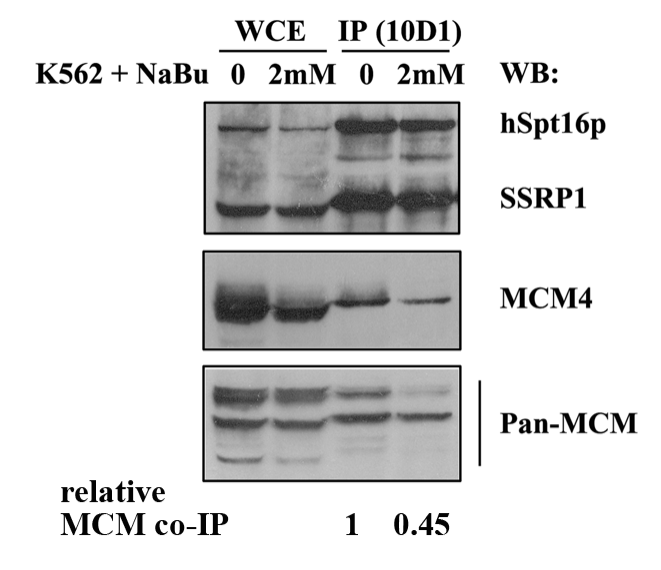
**The FACT-MCM interaction is reduced in response to growth inhibition**. Immunoblot showing the levels of FACT, MCM4, and MCMs in K562 cell extracts (lanes 1 & 2) and in 10D1 immunoprecipitates (lanes 3 & 4). Before extraction, K562 cells were either uninduced (lanes 1 & 3) or induced to differentiate with 3-day treatment of 2 mM sodium butyrate (NaBu) (lanes 2 & 4). The number at the bottom illustrates the relative levels of MCM co-immunoprecipitated by the 10D1 antibody. The degree of co-IP ("relative MCM co-IP"), expressed as a ratio in band intensity of total MCM to FACT, was normalized to the control treatment group.

Near lack of overlapping signals between FACT and MCM at the G_0_/early G_1 _stage of cell cycle led to the speculation that the FACT-MCM interaction may be positively regulated by cell proliferation. To entertain this possibility, we examined the FACT-MCM complex formation in quiescent cells. Interestingly, consistent with the localization data, we detected less MCM proteins (~45% of control) in the FACT immunoprecipitates in K562 cells upon entry into a differentiation/G_0_-like state (induced by treatment with sodium butyrate). This suggests that the formation/disruption of the FACT-MCM complex is correlated with cell proliferation (Figure 6).

## Discussion

**Figure 7:**
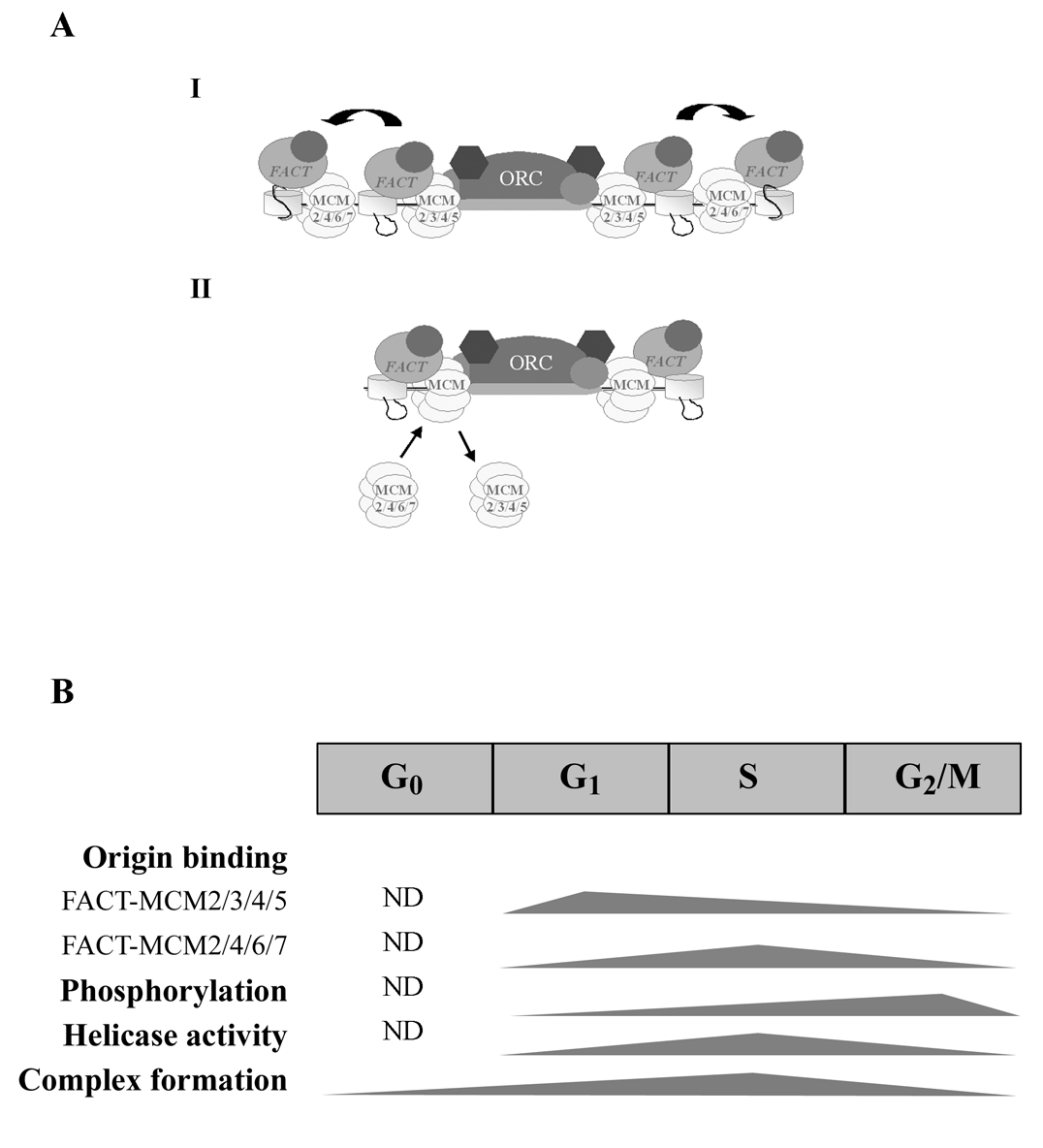
**Hypothetical paradigms of the regulatory mechanisms underlying the functional cooperation between FACT and MCM complexes**. (A) Putative models for the distinct roles of the two identified FACT-MCM complexes. As shown in Figure 3, the two assemblies may be recruited to the origin region in a temporal fashion, with FACT-MCM2/3/4/5 preceding FACT-MCM2/4/6/7. Both complexes are enzymatically active, and they may exert their activities, in two possible modes (I & II), at distinct and/or sequential stages during the early phase of DNA replication initiation (see Discussion). (B) The FACT-MCM complex constitutes an important activity that facilitates chromatin DNA unwinding during DNA replication [29]. Functional attributes of the FACT-MCM complexes are coordinated with the cell cycle. The temporal mode of such regulation is depicted in this diagram, in which the relative levels of the indicated functional attributes are represented. Origin association of the two FACT-MCM complexes reflects their distinct roles during origin establishment and replication initiation (Figure 3). DNA helicase activity and protein mitotic phosphorylation are inversely regulated to ensure proper integration and resetting of the complex activity within the cell cycle (Figures 4 & 5). Finally, the formation of the FACT-MCM complex is associated with cell proliferation and S phase (Figure 6). ND, characteristics of the indicated functional attributes in the quiescent state (or upon exit from cell cycle) have not been determined.

In this report, we provide several lines of evidence describing that cell cycle-associated signaling that governs normal S phase progression accounts for a major regulatory mechanism for the functional interaction between FACT and MCM complexes. We demonstrate that, during the period following pre-RC assembly and continuing into S phase, origin recruitment and function of the two types of complex is distinct. Furthermore, we show that the phosphorylation profile of the FACT-associated MCM4 undergoes a cell cycle-dependent change, which is directly correlated with the catalytic activity of the FACT-MCM helicase complex. At the quaternary structure level, physical interaction between FACT and MCM complexes is generally dependent on persistent cell cycle and further stabilized upon S phase entry. Thus, like the other proteins essential for DNA synthesis, the FACT-MCM-associated chromatin unwinding activity is tightly controlled at various levels by the cell cycle regulation (Figure 7B). Proper function, as well as timely down-regulation, of these complexes is necessary for maintaining once-per-cycle DNA replication and euploid gene balance.

We made an interesting and novel finding of the existence of multiple assemblies of the FACT-MCM complex: FACT-MCM2/4/6/7 and FACT-MCM2/3/4/5. Based on the previous observations on the stable formation of different subassemblies among the six MCM subunits [15-17,21], we initially postulated that the identified FACT-MCM2/3/4/5 complex may serve as a regulator to the presumably "catalytic" assembly of FACT-MCM2/4/6/7. However, we subsequently detected DNA unwinding activities in both of the immunocomplexes. Despite this discrepancy, which may be attributed to the intrinsic difference between the endogenous (as in FACT-MCM) and the reconstituted complexes, the true architecture as well as the catalytic properties of the MCM complexes in the cellular context is still largely unknown. The existence of multiple DNA helicase subcomplexes (or the FACT-MCM subcomplexes) has several explanations. First, certain subcomplexes still may serve as regulators to other catalytic counterparts in the cell, as proposed previously. Such coordinated action potentially contributes to the highly regulated process of DNA duplication. Second, combinatorial association of subunits may serve as the underlying basis for differential origin selection (early vs. late). To this end, further functional elucidation of these complexes possibly will rely on better knowledge of mammalian origin organization and techniques such as high-resolution ChIP. Third, these complexes could differentially act at discrete steps during DNA synthesis (initiation vs. elongation, or early initiation vs. late initiation). Based on the data presented in Figure 3, which distinguish the pre-RC association and temporal mode of origin binding between the two subcomplexes, we favor this paradigm (Figure 7A). Our results are consistent with a model in which the FACT-MCM2/3/4/5 is first recruited to the origin at the stage of pre-RC formation and origin establishment. Its helicase activity may be responsible for local, albeit partial, chromatin unwinding. Upon pre-RC assembly and entry into S phase, FACT-MCM2/4/6/7 subsequently becomes associated with the origin region, either juxtaposed to the already bound FACT-MCM2/3/4/5 (Figure 7A, model I), or substituting its occupancy (model II). It may then be involved in unwinding the origin to a more global extent and facilitating the association of additional factors and establishment of replication fork. Importantly, our results provide a plausible explanation for the hitherto uncharacterized roles of multiple MCM subcomplexes. The dynamics of spatial and temporal distribution of these FACT-MCM subcomplexes is an important research subject in the future.

The cell cycle-dependent manner through which FACT and MCM physically and functionally interact with each other was demonstrated by immunostaining analysis (Figure 5B) and immunocomplex helicase assay (Figure 5A). The observed behaviors of the FACT-associated MCM4 recall the mitotic-specific hyperphosphorylation and functional downregulation of MCM4, as reported previously [11]. Such mode of regulation, presumably through a conserved mechanism, suggests the involvement of cell cycle regulators such as Cdk2/cyclin A or cyclin B [10-13]. In addition to catalytic inactivation, mitotic hyperphosphorylation of MCM4 may concomitantly lead to dissociation from chromatin, as indicated by these reports. Additionally, in accordance with the finding by Ishimi and Komamura-Kohno [11], we observed a moderate but reproducible increase in catalytic activity of the FACT-MCM complex isolated from the G_1_/S-synchronized cells as compared to those at other phases (Figure 5A). Concurrently, there was a greater extent of mobility shift of MCM4 in the S phase immunocomplex as compared to that of asynchronous cells (lanes 1 and 2, Figure 4B). However, it is unclear at present whether or which signaling pathway underlies such modification and catalytic activation. Cdc7/Dbf4 (DDK) complex is a likely candidate kinase regulator [2]. Identification of these regulators may be a future research subject, and understanding of this signaling pathway will aid in further characterization of FACT-MCM or MCM proteins in general. Taken together, the consequences of these phosphorylation events reflect the critical integration of replication with cell cycle as well as a temporal resetting of MCM activity.

## Competing interests

The authors declare that they have no competing interests.

## Authors' contributions

BCMT, HL, and CLL carried out the experiments. BCMT and SCL conceived of the study, and participated in its design and coordination and helped to draft the manuscript.

## Supplementary Material

Additional file 1 **Supplementary Figures S1A, B, C, D**. This file contains figures and legends describing additional experiments that serve as supplementary information for the manuscript. Click here for file
