# Supplementary material for: Functional cooperation between FACT and MCM is coordinated with cell cycle and differential complex formation
Source: J Biomed Sci. 2010 Feb 16;17(1):11. doi: 10.1186/1423-0127-17-11 (PMC2848000; doi:10.1186/1423-0127-17-11)

Supplementary Information (Tan et al.)

Figure S1 Legends

Figure S1A

To exclude the possibility that the FACT antibodies cross-react with the MCM proteins, we checked whether the 10D1 antibody could non-specifically pull down MCMs. To this end, HeLa cell extracts were prepared from control and *SSRP1*^{RNAi} cells ("Input", which illustrates the depletion of SSRP1). Immunoprecipitation was then done with the 10D1 antibody. Immunoblotting experiments using antibodies against SSRP1, β-actin, MCM2, and MCM4 subsequently demonstrate that, in the context of SSRP1 down-regulation, the levels of MCM co-immunoprecipitation were also proportionally reduced, thus strengthening the specificity of the FACT antibodies.

Figure S1B

This figure demonstrates the extent of MCM3 and MCM4 depletion (by RNAi) in the samples for the helicase assay shown in Fig. 1D. HeLa cell extracts were prepared from control, *MCM4*^{RNAi}, or *MCM3*^{RNAi} cells, and subjected to immunoblotting using the indicated antibodies.

Figure S1C

Cell cycle synchronization of the cells used in Figure 3B was analyzed based on DNA content by flow cytomery. Stages of cell cycle, corresponding to those in Figure 3B, are indicated on the left.

Figure S1D

Western blotting analysis of the levels of MCM2/4 in the 10D1 and 8D2 immunocomplexes used for the helicase assay shown in Figure 4A. These data demonstrate that there were equivalent amounts of the helicase among the different immunoprecipitation samples.

Supplementary Figure S1 (Tan et al.)

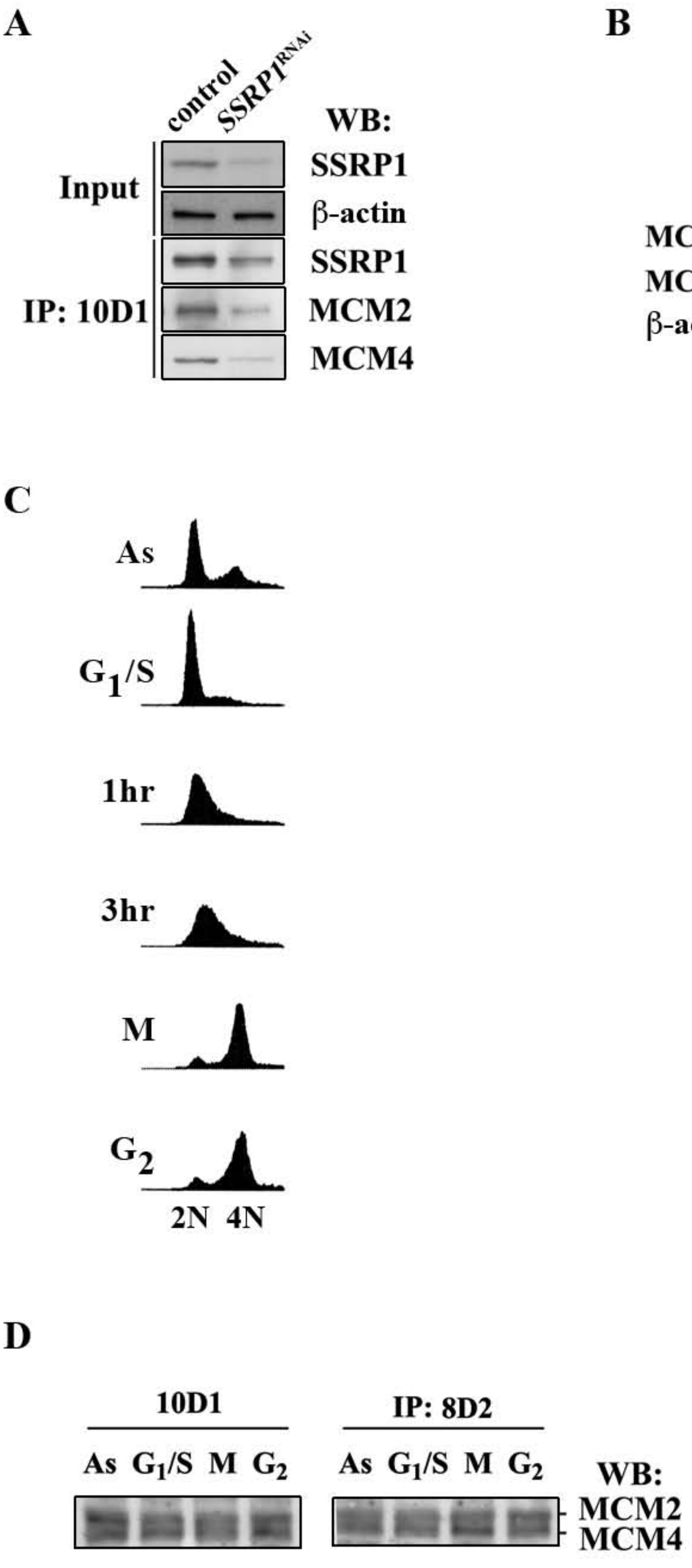

Supplement: Additional file 1 — Supplementary Figures S1A, B, C, D. This file contains figures and legends describing additional experiments that serve as supplementary information for the manuscript. [file 1423-0127-17-11-S1.PDF]
